# Current landscape of minimally invasive pancreatectomy for neoplasms: A retrospective cohort study

**DOI:** 10.1002/wjs.12408

**Published:** 2024-11-22

**Authors:** Rejoice F. Ngongoni, Busisiwe Mlambo, I‐Fan Shih, Yanli Li, Sherry M. Wren

**Affiliations:** ^1^ Department of Surgery Stanford University School of Medicine Stanford California USA; ^2^ Intuitive Surgical Sunnyvale California USA; ^3^ Department of Surgery Stanford University VA Palo Alto Care System Palo Alto California USA

**Keywords:** laparoscopic, minimally invasive, non‐pancreaticoduodenectomy, pancreatectomy, pancreaticoduodenectomy, robotic

## Abstract

**Background:**

To evaluate recent minimally invasive pancreatectomy (MIP) trends for neoplastic disease and compare perioperative outcomes.

**Methods:**

Patients who underwent open (OS) or MIP (laparoscopic‐LS or robotic‐RS) pancreaticoduodenectomy (PD) or non‐pancreati‐coduodenectomy resections (non‐PD) were identified from PINC AI Healthcare Database. Outcomes were compared using multivariable regressions.

**Results:**

OS was the predominant approach for PD (87.8%); MIP was more common in non‐PD (48.5%) than PD with a substantial RS uptake (11.7%–29.9%). In PDs, outcomes were similar except OS had a longer length of stay (LOS) and lower costs. In non‐PDs, MIP patients were less likely to have prolonged LOS, intensive care unit admission, and overall complications than OS. Conversion to OS was lower in the RS approach than LS in PD and non‐PD.

**Conclusions:**

MIP for non‐PD has become the most common operative approach with improved outcomes; MIP‐PD has flat adoption and similar outcomes to OS. Robotics facilitates MIP (PD and non‐PD) completion through fewer conversions to open surgery (OS).

## 1 INTRODUCTION

The surgical approach for pancreatic resection for neoplasm is based on anatomic tumor location. Pancreatic head lesions are treated with pancreaticoduodenectomy (PD) and body and tail tumors by non‐pancreaticoduodenectomy resections (non‐PD). Moreover, resections can be performed via open or minimally invasive approaches further categorized as laparoscopic (LS) or robotic (RS) procedures. Reported benefits of minimally invasive pancreatectomy (MIP) compared to open surgery (OS) include decreased blood loss, length of stay (LOS), recovery times and complications, and improved cost‐effectiveness and long‐term cosmetic satisfaction of patients. Clinical equipoise, as measured by overall quality of life, is comparable between the two approaches.^1^, ^2^, ^3^

The adoption of MIP has been gradual. The proportion of minimally invasive non‐pancreaticoduodenectomy (MIS non‐PD) slowly increased in the USA from 2.4% to 7.8% from 1998 to 2009.^4^, ^5^ In Norway, 59% of non‐PDs were performed minimally invasively between 2012 and 2016.^5^ A study in the USA showed that among minimally invasive pancreaticoduodenectomy (MIPD), robotic PD for pancreatic adenocarcinoma increased from 12.8% to 34.6% between 2010 and 2020.^6^ Technological improvements such as energy, improved image quality, and development of the robotic platform have supported the application of minimally invasive techniques to complex operations. However, national data on the proportion of PDs that are performed minimally invasively in the USA are minimal.

Multiple retrospective studies have demonstrated the feasibility and safety of MIP when compared to the open approach.^7^, ^8^, ^9^, ^10^, ^11^ Although oncologic outcomes initially appeared to be inferior to the open technique, subsequent studies have shown acceptable equivalent oncologic and survival outcomes between the two approaches, with some studies showing superior oncologic outcomes with MIP approaches.^12^, ^13^ Additionally, the procedure cost of MIP has been reportedly higher than the open approach. However, with reduced LOS, lower hospital costs have been associated with MIP in Europe and the USA.^14^, ^15^

As the field evolves, evaluating MIP adoption and the outcomes is important to inform management decisions. This study aims to report (a) recent trends in the adoption of minimally invasive pancreatectomy (laparoscopic or robotic) for neoplasms in the USA, (b) postoperative clinical outcomes among open, laparoscopic, and robotic approaches for PD and non‐PD resections, and (c) costs associated with each approach. We hypothesize that adoption of minimally invasive pancreatectomy is increasing, and that MIP has superior outcomes and reduced total costs when compared to open pancreatectomy.

## 2 MATERIALS AND METHODS

### 2.1 Data source

This retrospective study uses the PINC AI Healthcare Database (PHD), formerly known as Premier Healthcare Database, an extensive US hospital‐based, service‐level, and all‐payer database containing inpatient discharge data.^16^ Hospitals provide administrative, healthcare utilization, and financial information from patient encounters. The PHD captures approximately 25% of US inpatient admissions from more than 740 hospitals located in all the US Census geographic divisions annually. Patients can be followed in the same hospital across inpatient and hospital‐based outpatient settings over time. PHD adheres to strict quality control where only 0.01% of data are missing for key categories. As data within the PHD are deidentified and compliant with the Health Insurance Portability and Accountability Act privacy rules, institutional review board approval was exempted and no patient consent was required for the study. The study was reported according to the STROBE guidelines.^17^

### 2.2 Study population

**FIGURE 1:**
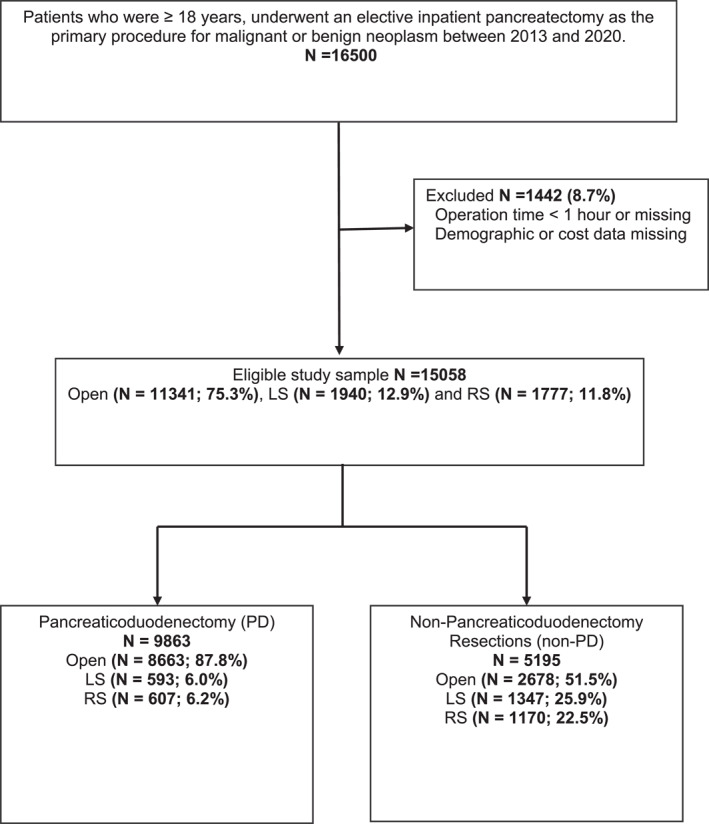
Study flowchart. LS, laparoscopic surgery; RS, robotic surgery.

Patients aged ≥18 years who underwent an elective, inpatient PD, or non‐PD as a primary procedure for benign or malignant neoplasm between January 1, 2013, and December 31, 2020, were included in the study. Each pancreatectomy group was further classified into open (OS), laparoscopic (LS), or robotic‐assisted (RS) groups based on the International Classification of Diseases, 9th and 10th Revisions Procedure classification system (ICD‐9‐PCS/ICD‐10‐PCS codes), Current Procedural Terminology (CPT) codes, and billing text fields (Supplementary Table 1 in Supporting Information S1). Laparoscopic or robotic cases converted to open were counted as intention‐to‐treat by the originally planned surgical approach. Patients were excluded if they had operative time <1 h, no operative time data, or had missing demographic, hospital, and cost information (Figure 1).

### 2.3 Outcome measures

Outcomes for the index hospital encounter included hospital length of stay (LOS), prolonged LOS, conversion rates to open surgery, splenectomy, operating room (OR) time, intensive care unit (ICU) admission, in‐hospital mortality, discharge to home rates, in‐hospital reoperations, and complications. Prolonged LOS was defined as ≥14 days and ≥10 days for the PD and non‐PD groups, respectively.^18^, ^19^, ^20^, ^21^ The index encounter was defined as the hospital encounter in which a patient underwent pancreatectomy. ICD codes were used to identify in‐hospital complications, including intraoperative, pulmonary, mechanical ventilation, bleeding, blood transfusion, infections, and venous thromboembolism. We assessed 30‐day readmission to the same hospital and perioperative hospital costs for service utilization. Perioperative cost is the sum of the index hospitalization cost and any additional costs incurred within 30 days of discharge from the same hospital for each patient. Each hospital uses cost accounting models and systems to determine procedural costs. Healthcare systems that submit ratios of costs to charges also provide charge data to PINC AI. PINC AI and the hospitals ascribe Medicare cost to charge ratios and then PHD validates all costs and charges. Additional outcomes included the proportion of cases performed via open, laparoscopic, and robotic approaches in a given year. We also determined the percentage of high‐volume versus low‐volume centers completing MIS‐PD and MIS non‐PD cases. For PD cases, hospitals were considered high‐volume centers if ≥ 9 cases were performed yearly and low‐volume if < 9 cases were performed yearly.^22^, ^23^ For non‐PD cases, hospitals were defined as high‐volume if ≥ 5 cases were performed per year and low‐volume non‐PD centers if < 5 cases per year were performed.^24^, ^25^

### 2.4 Study covariates

Patient and hospital characteristics during the index hospital admission were identified. Patient‐level characteristics included age, sex, race, payor type, primary diagnosis (malignant or benign neoplasm), body mass index (BMI), and noncancer Charlson comorbidity index (CCI) scores. Hospital characteristics included geographic region, urban/rural area, teaching status, and bed size.

### 2.5 Statistical analysis

Summary statistics were reported as means and standard deviations (SDs) and compared using the Student's *t*‐test or analysis of variance (ANOVA) for normally distributed continuous variables. Abnormally distributed continuous variables were compared using the Mann–Whitney *U* test and reported as medians with interquartile range. Categorical variables were compared using the chi‐squared test and reported as percentages. Adoption trends were plotted by year and reported as annual volumes and proportions for each surgical approach. Fold change for each surgical approach over time was calculated by dividing the proportional use of the approach in 2020 with its proportional use in 2013. Multivariable logistic regression and gamma regression with identity link models were used to compare outcomes between operative approaches while controlling for baseline patient and hospital characteristics (study covariates) among the groups. The odds ratio (OR)/mean difference (MD) and 95% confidence interval (CI) were calculated and considered statistically significant if *p* < 0.05. All analyses were performed using the *R* software version 4.1.1.

## 3 RESULTS

### 3.1 Baseline patient and hospital characteristics

The study cohort (*n* = 15,058) was largely composed of patients who were White (73.0%), aged ≥65 years (57.8%), and had malignant neoplasm as the primary diagnosis (82.4%). Table 1 shows baseline characteristics for patients who underwent open, laparoscopic, and robotic pancreatectomy.

**TABLE 1:** Baseline patient and hospital‐related characteristics of patients who underwent pancreatectomy.

	Pancreaticoduodenectomy (PD)					Non‐pancreaticoduodenectomy (Non‐PD) resections				
Variable	Overall, *N* = 9863	Type of procedure				Overall, *N* = 5195	Type of procedure			
		OS, *N* = 8663	LS, *N* = 593	RS, *N* = 607	*P* Value		OS, *N* = 2678	LS, *N* = 1347	RS, *N* = 1170	*P* Value
Age					0.130					0.430
18–44	371 (3.8)	330 (3.8)	16 (2.7)	25 (4.1)		501 (9.6)	260 (9.7)	139 (10.3)	102 (8.7)	
45–65	3613 (36.6)	3163 (36.5)	242 (40.8)	208 (34.3)		1867 (35.9)	985 (36.8)	466 (34.6)	416 (35.6)	
65+	5879 (59.6)	5170 (59.7)	335 (56.5)	374 (61.6)		2827 (54.4)	1433 (53.5)	742 (55.1)	652 (55.7)	
Sex					0.930					0.850
Female	4705 (47.7)	4133 (47.7)	286 (48.2)	286 (47.1)		2896 (55.7)	1503 (56.1)	747 (55.5)	646 (55.2)	
Male	5158 (52.3)	4530 (52.3)	307 (51.8)	321 (52.9)		2299 (44.3)	1175 (43.9)	600 (44.5)	524 (44.8)	
Race					0.001					0.001
African American	990 (10.0)	886 (10.2)	54 (9.1)	50 (8.2)		598 (11.5)	322 (12.0)	127 (9.4)	149 (12.7)	
Hispanic	683 (6.9)	610 (7.0)	39 (6.6)	34 (5.6)		404 (7.8)	198 (7.4)	125 (9.3)	81 (6.9)	
Caucasian	7253 (73.5)	6312 (72.9)	452 (76.2)	489 (80.6)		3740 (72.0)	1897 (70.8)	1000 (74.2)	843 (72.1)	
Others	937 (9.5)	855 (9.9)	48 (8.1)	34 (5.6)		453 (8.7)	261 (9.7)	95 (7.1)	97 (8.3)	
Payer					0.029					0.070
Commercial	3031 (30.7)	2636 (30.4)	193 (32.5)	202 (33.3)		1826 (35.1)	930 (34.7)	469 (34.8)	427 (36.5)	
Medicaid	612 (6.2)	550 (6.3)	43 (7.3)	19 (3.1)		344 (6.6)	193 (7.2)	95 (7.1)	56 (4.8)	
Medicare	5750 (58.3)	5059 (58.4)	333 (56.2)	358 (59.0)		2779 (53.5)	1417 (52.9)	721 (53.5)	641 (54.8)	
Others	470 (4.8)	418 (4.8)	24 (4.0)	28 (4.6)		246 (4.7)	138 (5.2)	62 (4.6)	46 (3.9)	
Primary diagnosis					<0.001					0.150
Benign	1023 (10.4)	911 (10.5)	36 (6.1)	76 (12.5)		1631 (31.4)	815 (30.4)	423 (31.4)	393 (33.6)	
Malignant	8840 (89.6)	7752 (89.5)	557 (93.9)	531 (87.5)		3564 (68.6)	1863 (69.6)	924 (68.6)	777 (66.4)	
BMI					0.970					0.440
Normal	7083 (71.8)	6217 (71.8)	430 (72.5)	436 (71.8)		3950 (76.0)	2030 (75.8)	1036 (76.9)	884 (75.6)	
Obese	506 (5.1)	441 (5.1)	29 (4.9)	36 (5.9)		384 (7.4)	183 (6.8)	101 (7.5)	100 (8.5)	
Overweight	1068 (10.8)	942 (10.9)	64 (10.8)	62 (10.2)		590 (11.4)	320 (11.9)	141 (10.5)	129 (11.0)	
Underweight	1206 (12.2)	1063 (12.3)	70 (11.8)	73 (12.0)		271 (5.2)	145 (5.4)	69 (5.1)	57 (4.9)	
CCI score					0.008					0.120
0	4699 (47.6)	4154 (48.0)	257 (43.3)	288 (47.4)		2907 (56.0)	1455 (54.3)	772 (57.3)	680 (58.1)	
1	1297 (13.2)	1121 (12.9)	75 (12.6)	101 (16.6)		739 (14.2)	383 (14.3)	192 (14.3)	164 (14.0)	
2+	3867 (39.2)	3388 (39.1)	261 (44.0)	218 (35.9)		1549 (29.8)	840 (31.4)	383 (28.4)	326 (27.9)	
Region					<0.001					<0.001
Midwest	1655 (16.8)	1431 (16.5)	84 (14.2)	140 (23.1)		884 (17.0)	460 (17.2)	170 (12.6)	254 (21.7)	
Northeast	1827 (18.5)	1634 (18.9)	124 (20.9)	69 (11.4)		925 (17.8)	474 (17.7)	294 (21.8)	157 (13.4)	
South	4889 (49.6)	4223 (48.7)	283 (47.7)	383 (63.1)		2679 (51.6)	1306 (48.8)	714 (53.0)	659 (56.3)	
West	1492 (15.1)	1375 (15.9)	102 (17.2)	15 (2.5)		707 (13.6)	438 (16.4)	169 (12.5)	100 (8.5)	
Hospital location					<0.001					0.013
Rural	555 (5.6)	507 (5.9)	36 (6.1)	12 (2.0)		268 (5.2)	148 (5.5)	79 (5.9)	41 (3.5)	
Urban	9308 (94.4)	8156 (94.1)	557 (93.9)	595 (98.0)		4927 (94.8)	2530 (94.5)	1268 (94.1)	1129 (96.5)	
Teaching hospital	7354 (74.6)	6477 (74.8)	416 (70.2)	461 (75.9)	0.032	3771 (72.6)	1902 (71.0)	1030 (76.5)	839 (71.7)	<0.001
Bed size					<0.001					<0.001
0–299	859 (8.7)	768 (8.9)	71 (12.0)	20 (3.3)		527 (10.1)	299 (11.2)	137 (10.2)	91 (7.8)	
300–399	1416 (14.4)	1298 (15.0)	91 (15.3)	27 (4.4)		690 (13.3)	394 (14.7)	190 (14.1)	106 (9.1)	
400–499	1302 (13.2)	1145 (13.2)	95 (16.0)	62 (10.2)		664 (12.8)	365 (13.6)	151 (11.2)	148 (12.6)	
500+	6286 (63.7)	5452 (62.9)	336 (56.7)	498 (82.0)		3314 (63.8)	1620 (60.5)	869 (64.5)	825 (70.5)	

Abbreviations: BMI, body mass index; CCI, Charlson comorbidity index; LS, laparoscopic surgery; OS, open surgery; PD, pancreaticoduodenectomy; RS, robotic‐assisted surgery.

### 3.2 Temporal trends of surgical approaches

**FIGURE 2:**
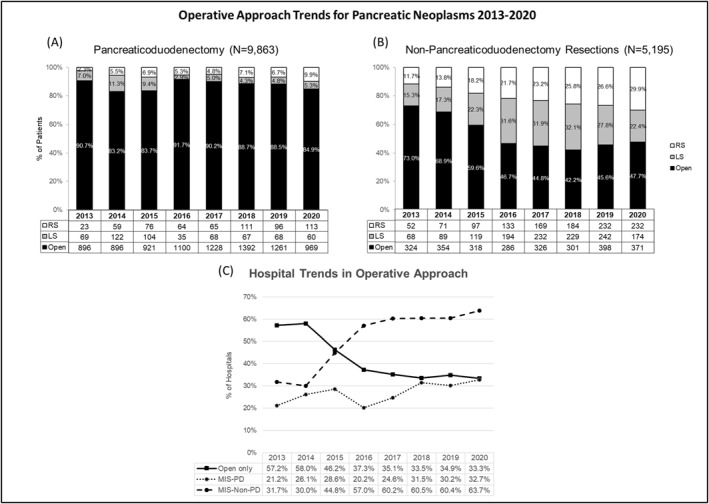
Trend utilization of an operative approach for pancreatectomy patients with pancreatic neoplasms (A) pancreaticoduodenectomy, (B) non‐pancreaticoduodenectomy resections, and (C) utilization of open and minimally invasive pancreatectomy among hospitals. MIS non‐PD, minimally invasive non‐pancreaticoduodenectomy; MIS‐PD, minimally invasive pancreaticoduodenectomy; LS, laparoscopic; RS, robotic.

A total of 15,058 patients underwent pancreatectomy (PD = 9863 and non‐PD = 5195) for neoplasms between 2013 and 2020 (Figure 1). Among PD resections, the open approach was predominant. MIPD (LS and RS) was less commonly performed, with adoption rates fluctuating between 8.2% and 16.8% over the study period (Figure 2A). In non‐PD resections, MIP non‐PD rates were higher than PD; MIP non‐PD became the most common approach in 2016 (53.3%; Figure 2B). Overall, RS had substantial uptake and increased 4.3‐fold from 2.3% to 9.9% for PD and 2.6‐fold from 11.7% to 29.9% for non‐PD over 8 years.

### 3.3 Hospital volume trends

In the early years, over half of the hospitals only performed open pancreatectomy procedures in their facilities (2013: 57.2% and 2014: 58.0%) as shown in Figure 2C. The percentage decreased over subsequent years and only a third of hospitals did not perform MIP by 2020 (Figure 2C). This was mainly driven by the proportion of non‐PD hospitals doubling from 31.7% to 63.7% over 8 years. In contrast, the hospital uptake of minimally invasive PD (MIPD) was modest from 21.2% to 32.7%. When we stratified by hospital pancreatectomy volume (Supplementary Table 2 in Supporting Information S1), there was an increase in both low‐ and high‐volume hospitals that performed MIP over the study period. High‐volume hospitals increased in proportion from 42.9% to 66.0% for PD and 80.8%–95.0% for non‐PD, whereas the proportion of low‐volume hospitals (<5 cases/year) that performed non‐PD cases almost doubled from 34.1% to 65.0%.

### 3.4 Comparison of perioperative and postoperative outcomes

#### 3.4.1 PD resections

Patients with MIPD resections did not have many significantly different outcomes than OS. LS had longer operative time (mean difference 41 min) and a 36% increased risk of blood transfusion (Table 2). RS had an increased operative time (mean difference 107 min), a 26% increased risk of transfusion, and a shorter LOS (mean difference −0.86, *p* < 0.001; 11.3 vs. 10.2 days). The 30‐day perioperative costs for MIP were significantly higher than OS (LS: mean difference $4186, *p* = 0.015; RS: mean difference $9977, *p* < 0.001) (Table 2). When compared RS to LS, RS had lower risk of conversion to open (OR 0.30 [0.23–0.40] and *p* < 0.001), lower risk for ICU admission (OR 0.30 [0.23–0.40] and *p* < 0.001), and trended toward higher perioperative costs than the LS group (mean difference $4691, *p* = 0.052; Supplementary Table 3 in Supporting Information S1).

**TABLE 2:** Multivariable analysis of association of surgical approach and postoperative outcomes for pancreaticoduodenectomy (PD).

Variable	Type of procedure				LS versus Open		RS versus Open	
	OS, *N* = 8663	LS, *N* = 593	RS, *N* = 607	*P* Value	Diff/OR (95% CI)	*P* Value	Diff/OR (95% CI)	*P* Value
LOS				<0.001				
Mean (SD)	11.3 (9.5)	11.2 (9.2)	10.2 (10.9)		0.09 (−0.63 to 0.88)	0.810	−0.86 (−1.5 to −0.16)	0.012
Median (Q1, Q3)	8 (6, 13)	8 (6, 13)	7 (5, 11)					
LOS ≥14 days	2032 (23.5%)	141 (23.8%)	100 (16.5%)	<0.001	1.01 (0.82 to 1.23)	0.940	0.66 (0.53 to 0.82)	<0.001
OR time				<0.001				
Mean (SD)	440.0 (350.0)	479.6 (170.2)	530.4 (199.9)		41 (19 to 65)	<0.001	107 (81 to 134)	<0.001
Median (Q1, Q3)	407 (330, 510)	465 (360, 555)	480 (405, 594)					
ICU admission	5499 (63.5%)	357 (60.2%)	371 (61.1%)	0.16	0.88 (0.74 to 1.05)	0.160	0.88 (0.74 to 1.05)	0.151
In‐hospital mortality	248 (2.9%)	21 (3.5%)	18 (3.0%)	0.63	1.19 (0.73 to 1.84)	0.456	1.02 (0.60 to 1.63)	0.932
Discharge home	6994 (80.7%)	470 (79.3%)	482 (79.4%)	0.52	0.92 (0.74 to 1.14)	0.431	0.93 (0.75 to 1.16)	0.508
Index reoperation	457 (5.3%)	39 (6.6%)	40 (6.6%)	0.17	1.23 (0.86 to 1.71)	0.227	1.34 (0.94 to 1.87)	0.091
In‐hospital complication	3999 (46.2%)	296 (49.9%)	285 (47.0%)	0.20	1.16 (0.98 to 1.37)	0.093	1.00 (0.85 to 1.19)	0.972
Intraoperative	182 (2.1%)	20 (3.4%)	15 (2.5%)	0.11	1.54 (0.93 to 2.41)	0.073	1.26 (0.70 to 2.09)	0.411
Pulmonary	1123 (13.0%)	83 (14.0%)	99 (16.3%)	0.054	1.08 (0.84 to 1.37)	0.540	1.27 (1.00 to 1.59)	0.045
Mechanical ventilation	638 (7.4%)	57 (9.6%)	50 (8.2%)	0.11	1.31 (0.97 to 1.74)	0.068	1.16 (0.84 to 1.56)	0.362
Transfusion	1674 (19.3%)	149 (25.1%)	136 (22.4%)	<0.001	1.36 (1.11 to 1.65)	0.002	1.26 (1.02 to 1.54)	0.026
Bleeding	2284 (26.4%)	172 (29.0%)	174 (28.7%)	0.19	1.17 (0.97 to 1.40)	0.107	1.05 (0.87 to 1.27)	0.597
Infections	1243 (14.3%)	90 (15.2%)	86 (14.2%)	0.85	1.06 (0.83 to 1.33)	0.634	1.00 (0.78 to 1.26)	0.989
Venous thromboembolism	180 (2.1%)	13 (2.2%)	14 (2.3%)	0.92	1.01 (0.54 to 1.73)	0.964	1.12 (0.61 to 1.90)	0.680
30‐day readmission	1592 (18.4%)	103 (17.4%)	123 (20.3%)	0.40	0.96 (0.76 to 1.19)	0.689	1.09 (0.88 to 1.34)	0.434
30‐day perioperative cost				<0.001				
Mean (SD)	48,039 (40,936)	51,063 (47,613)	54,381 (44,067)		4186 (987 to 7654)	0.015	8877 (5492 to 12,541)	<0.001
Median (Q1, Q3)	37,330 (27,847, 53,371)	38,061 (29,192, 55,651)	43,083 (32,760, 58,189)					

Abbreviations: CI, confidence interval; Diff, mean difference; ICU, intensive care unit; LOS, length of stay; LS, laparoscopic surgery; OR, odds ratio; OR time, operative room time; OS, open surgery; RS, robotic‐assisted surgery.

#### 3.4.2 Non‐PD resections

Overall, the MIP approach had improved outcomes compared to OS. Both LS had lower risk of ICU admission, in‐hospital complications, and decreased LOS. In addition, RS had lower risk for splenectomy (0.66 [0.57–0.76], *p* < 0.001) (Table 3). Compared to OS, 30‐day perioperative costs were lower and higher for LS and RS, respectively (LS: MD $−2066, *p* = 0.005 and RS: MD $5286, *p* < 0.001).

**TABLE 3:** Multivariable analysis of association of surgical approach and postoperative outcomes for non‐pancreaticoduodenectomy resections (Non‐PD).

Variable	Type of procedure				LS versus Open		RS versus Open	
	OS, *N* = 2678	LS, *N* = 1347	RS, *N* = 1170	*P* Value	OR/Diff (95% CI)	*P* Value	OR/Diff (95% CI)	*P* Value
LOS				<0.001				
Mean (SD)	7.4 (5.7)	6.2 (5.1)	6.1 (5.2)		−1.1 (−1.4 to −0.79)	<0.001	−1.2 (−1.5 to −0.86)	<0.001
Median (Q1, Q3)	6 (5, 8)	5 (4, 7)	5 (4, 7)					
LOS ≥10 days	435 (16.2%)	162 (12.0%)	111 (9.5%)	<0.001	0.71 (0.58 to 0.87)	<0.001	0.56 (0.44 to 0.70)	<0.001
OR time								
Mean (SD)	294.8 (195.1)	298.4 (155.2)	384.2 (188.9)	<0.001	6.2 (−4.4 to 17)	0.254	88 (74 to 102)	<0.001
Median (Q1, Q3)	267 (210, 345)	270 (210, 360)	339 (261, 450)					
Splenectomy	1816 (67.8%)	907 (67.3%)	691 (59.1%)	<0.001	0.98 (0.85 to 1.13)	0.764	0.66 (0.57 to 0.76)	<0.001
ICU Admission	1089 (40.7%)	353 (26.2%)	280 (23.9%)	<0.001	0.52 (0.44 to 0.60)	<0.001	0.45 (0.38 to 0.52)	<0.001
In‐hospital mortality	34 (1.3%)	10 (0.7%)	12 (1.0%)	0.31	0.59 (0.27 to 1.16)	0.146	0.80 (0.39 to 1.52)	0.508
Discharge home	2397 (89.5%)	1230 (91.3%)	1062 (90.8%)	0.15	1.29 (1.02 to 1.64)	0.035	1.16 (0.91 to 1.48)	0.250
Index reoperation	79 (2.9%)	30 (2.2%)	31 (2.6%)	0.41	0.75 (0.48 to 1.14)	0.191	0.97 (0.62 to 1.48)	0.887
In‐hospital complication	855 (31.9%)	320 (23.8%)	294 (25.1%)	<0.001	0.67 (0.58 to 0.79)	<0.001	0.72 (0.61 to 0.84)	<0.001
Intraoperative	48 (1.8%)	22 (1.6%)	16 (1.4%)	0.63	0.90 (0.53 to 1.49)	0.689	0.82 (0.44 to 1.43)	0.501
Pulmonary	234 (8.7%)	87 (6.5%)	85 (7.3%)	0.029	0.73 (0.56 to 0.95)	0.020	0.84 (0.64 to 1.10)	0.211
Mechanical ventilation	113 (4.2%)	44 (3.3%)	46 (3.9%)	0.34	0.77 (0.53 to 1.10)	0.157	0.95 (0.65 to 1.35)	0.760
Transfusion	333 (12.4%)	112 (8.3%)	92 (7.9%)	<0.001	0.63 (0.50 to 0.79)	<0.001	0.62 (0.48 to 0.79)	<0.001
Bleeding	473 (17.7%)	165 (12.2%)	173 (14.8%)	<0.001	0.67 (0.55 to 0.81)	<0.001	0.81 (0.67 to 0.99)	0.039
Infections	185 (6.9%)	72 (5.3%)	61 (5.2%)	0.050	0.78 (0.58 to 1.03)	0.088	0.73 (0.54 to 0.99)	0.045
Venous thromboembolism	32 (1.2%)	6 (0.4%)	11 (0.9%)	0.068	0.36 (0.13 to 0.81)	0.023	0.83 (0.40 to 1.63)	0.613
30‐day readmission	455 (17.0%)	216 (16.0%)	206 (17.6%)	0.56	0.93 (0.77 to 1.11)	0.403	1.04 (0.86 to 1.25)	0.676
30‐day perioperative cost				<0.001				
Mean (SD)	32,291 (26,240)	30,078 (23,585)	36,519 (33,743)		−2066 (−3493 to −611)	0.005	5286 (3547 to 7082)	<0.001
Median (Q1, Q3)	25,481 (18,793, 36,360)	23,819 (17,214, 33,571)	28,940 (21,633, 40,200)					

Abbreviations: CI, confidence interval; Diff, mean difference; ICU, intensive care unit; LOS, length of stay; LS, laparoscopic surgery; OR, odds ratio; OR time, operative room time; OS, open surgery; RS, robotic‐assisted surgery.

When comparing RS to LS, outcomes were similar except RS had longer OR time (mean difference: 82 min) and lower risk of splenectomy (OR 0.42 [0.33–0.54] and *p* < 0.001) (Supplementary Table 4 in Supporting Information S1).

Additionally, conversion to open surgery was less likely to occur in the RS group than the LS group (OR 0.42 [0.33–0.54] and *p* < 0.001). RS had higher 30‐day perioperative costs than LS (mean difference $7351 [5444 to 9291] and *p* < 0.001).

### 3.5 Comparison of MIP conversion to open versus nonconversion

The overall conversion rate was higher for MIPD than non‐PD (33.7% vs. 15.4%) and patients in the conversion groups had increased risk for complications and perioperative costs. The MIPD conversion group had higher risk for intraoperative complications (OR 2.11 [1.03–4.29] and *p* < 0.039) and transfusion (OR 1.43 [1.07–1.91] and *p* < 0.017) (Supplementary Table 5 in Supporting Information S1). On the other hand, the MIP non‐PD conversion group had higher risk for splenectomy (OR 3.24 [2.46–4.32] and *p* < 0.001), in‐hospital complications including transfusion (OR 2.94 [2.11–4.06] and *p* < 0.001), ICU admission (OR 2.47 [1.94–3.14] and *p* < 0.001), and increased LOS (mean difference 1.3 [0.71–1.9) and *p* < 0.001) compared to the nonconversion group (Supplementary Table 6 in Supporting Information S1). Perioperative costs between conversion and nonconversion groups were not significantly different in the MIPD group but were higher in the non‐PD group (mean difference $4946 (2061–8080) and *p* = 0.001).

## 4 DISCUSSION

This retrospective cohort study evaluated the recent trends in adopting minimally invasive pancreatectomy for pancreatic neoplasms in the United States from 2013 to 2020 in a large representative dataset of US hospitals. Since 2013, there has been an overall increase in MIP and the number of hospitals performing these cases. Both MIPD and non‐PD resections have seen increased adoption but at unequal rates. Non‐PD cases account for the greatest proportion of MIP procedures and are performed in two‐thirds of low pancreatectomy volume hospitals, which may be influenced by the fact that non‐PDs are technically more approachable for surgeons.^26^, ^27^, ^28^ Conversely, MIPD represents a more technically complex dissection coupled with multiple reconstructions. This could explain why 87.8% of PDs in this study are open and why MIPD has a slower adoption rate and is performed in fewer hospitals (∼25%).

The laparoscopic PD rate has been stagnant compared to robotic PD, which has seen a 4‐fold increase, even though the LS approach was first developed and had a reported easier learning curve.^26^, ^29^ In addition, the laparoscopic non‐PD rate has been diminishing compared to robotic non‐PD and there has been an almost 3‐fold increase in the number of robotic non‐PDs performed. Robotic pancreatectomy became the most common minimally invasive approach for both non‐PD (2016) and PD (2020), which could be due to surgeon‐perceived advantages of robotics, such as three‐dimensional visualization and increased dexterity, which facilitate dissection and reconstruction.^25^

Our study shows few advantages of MIPD over open approach. This reflects the literature where similar results include improved outcomes such as decreased LOS.^9^, ^10^, ^30^ However, a multi‐institutional study of eight centers showed open PD associated with improved outcomes, such as blood loss and major complications, compared to robotic PD.^31^ One possible explanation for this difference could be that this dataset more widely represents the current state of practice across the US instead of data from a small number of specialized centers. Our study showed no difference in mortality, which is consistent with other reports in the literature, but mortality results are mixed.^7^, ^32^, ^33^, ^34^ There is less data comparing robotic and laparoscopic approaches, but available data confirm that the approaches are similar except for the lower conversion rates with RS approach.^11^, ^35^, ^36^ We observed that the robotic PD group had a significantly lower incidence of conversion to open than LS. This is similar to the findings from the LAELAPS trials, which showed a robotic PD conversion of 6.5% (LAELAPS‐2) versus 11% for laparoscopic PD (LAELAPS‐3).^29^, ^35^, ^37^ Our study is in line with other studies that demonstrate longer OR times for LS than open surgery. This may be due to longer setup times and a steep learning curve of 39 cases for the robotic approach.^38^

In contrast to PD, MIP in non‐PD patients is associated with improved outcomes compared to OS including LOS, transfusions, ICU admission, and splenic preservation. The improved outcomes of the MIP non‐PD approach are likely multifactorial.^2^, ^3^, ^8^, ^12^, ^39^, ^40^ Despite longer OR times and costs, robotic non‐PD is advantageous and non‐inferior compared to OS and LS, respectively, consistent with other studies.^41^, ^42^ Additionally, robotic non‐PD had a lower conversion to open rate than laparoscopy 1:10 versus 1:5. This could be resultant of selection bias where surgeons choose patients with favorable anatomy such as small localized neoplasms for the robotic approach. The observation that robotic surgery displaces laparoscopy as the predominant MIS approach for non‐PD has accelerated in 2020 as more hospitals have robotic capabilities. If the observed trends continue, robotic surgery will overtake laparoscopy as the approach of choice despite increased OR times and costs.^41^, ^43^

Limitations of this study are related to the administrative dataset. PHD accounts for 25% of inpatient admissions and this could limit the generalizability of the results as some patient and surgeon populations may be underrepresented or overrepresented. When comparing the PHD to the American Hospital Association Dataset (AHAD), which includes all US hospitals, the rural–urban ratio is comparable (AHAD 76% vs. PHD 70%). PHD has a relatively similar representation of teaching hospitals (∼30% PHD vs. 40% AHAD).

In the USA, payer information can be used as a surrogate for demographic information. PHD is relatively similar to AHAD for Medicare/Medicaid (PHD 52% vs. AHAD 48%), private insurance (PHD 35% vs. AHAD 43%), and out‐of‐pocket (PHD 7% vs. AHAD 9%).^44^, ^45^ This study adjusted for various baseline patient and hospital characteristics in multivariable analysis to minimize selection bias. Unfortunately, in the USA, there is no uniform database for all healthcare encounters. PHD does not track patients across the entire health system and can only report data from hospital inpatient and outpatient facilities. Therefore, readmissions to non‐index facilities would not be captured. There is no data on surgeon experience or tumor and treatment‐specific information, which may influence the surgeon's choice of procedure and outcomes. This is still observational data; a randomized controlled trial would minimize bias and elucidate the differences among the three approaches.

## 5 CONCLUSIONS

MIP for non‐PD has become the most common operative approach with improved outcomes compared to OS. MIPD has flat adoption with similar outcomes to OS. Robotics facilitates completion of both PD and non‐PD minimally invasively through fewer conversions to OS. Robotic PD should be increasingly considered given non‐inferior outcomes to open surgery. Currently, there are ongoing randomized trials looking at minimally invasive pancreatectomy, which will offer high‐quality evidence and data. An unsolved challenge is that the USA observational cohort data may not represent results from European data because pancreatic surgery is not regionalized to designated centers, allowing individual hospitals and surgeons to choose the operative approach even when the pancreatectomy operative volume may be low. Regionalization of care in the USA might be necessary to ensure better and more uniform outcomes for complex procedures.

## AUTHOR CONTRIBUTIONS

**Rejoice F. Ngongoni**: Conceptualization, investigation, methodology, project administration, writing—original draft preparation, writing—review and editing. **Busisiwe Mlambo**: Conceptualization, writing—original draft, writing—review and editing. **I‐Fan Shih**: Formal analysis, methodology, writing—original draft, writing—review and editing. **Yanli Li**: Formal analysis, writing—review and editing. **Sherry M. Wren**: Conceptualization, investigation, methodology, supervision, writing—review and editing.

## CONFLICT OF INTEREST STATEMENT

Rejoice Ngongoni declares that she has no conflicts of interest. Busisiwe Mlambo is employed by and has stock ownership and stock options with Intuitive Surgical. I‐Fan Shih is employed by and has stock ownership and stock options with Intuitive Surgical. YanLi Li is employed by and has stock ownership and stock options with Intuitive Surgical. Sherry Wren is a consultant of and has stock ownership of Intuitive Surgical.

## ETHICS STATEMENT

Not needed.

## CONSENT FOR PUBLICATION

Not applicable. The manuscript does not contain data from any person.

## Supporting information

Supporting Information 1

## Data Availability

Research data are not shared.

## References

[1] Korrel, Maarten , Anne Roelofs , Jony van Hilst , Olivier R. Busch , Freek Daams , Sebastiaan Festen , Bas Groot Koerkamp , et al. 2021. “Long‐Term Quality of Life after Minimally Invasive vs Open Distal Pancreatectomy in the LEOPARD Randomized Trial.” Journal of the American College of Surgeons 233(6): 730–739.e9. 10.1016/j.jamcollsurg.2021.08.687. 34530127

[2] Björnsson, B. , A. Lindhoff Larsson , C. Hjalmarsson , T. Gasslander , and P. Sandström . 2020. “Comparison of the Duration of Hospital Stay after Laparoscopic or Open Distal Pancreatectomy: Randomized Controlled Trial.” British Journal of Surgery 107(10): 1281–1288. 10.1002/bjs.11554. 32259297

[3] Zhang, X.‐Feng , Alexandra G. Lopez‐Aguiar , George Poultsides , Eleftherios Makris , Flavio Rocha , Zaheer Kanji , Sharon Weber , et al. 2019. “Minimally Invasive versus Open Distal Pancreatectomy for Pancreatic Neuroendocrine Tumors: An Analysis from the U.S. Neuroendocrine Tumor Study Group.” Journal of Surgical Oncology 120(2): 231–240. 10.1002/jso.25481. 31001868

[4] Tran Cao, Hop S. , Nicole Lopez , David C. Chang , Andrew M. Lowy , Michael Bouvet , Joel M. Baumgartner , Mark A. Talamini , and Jason K. Sicklick . 2014. “Improved Perioperative Outcomes with Minimally Invasive Distal Pancreatectomy: Results from a Population‐Based Analysis.” JAMA Surg 149(3): 237–243. 10.1001/jamasurg.2013.3202. 24402232 PMC4383084

[5] Søreide, Kjetil , Frank Olsen , Linn S. Nymo , Dyre Kleive , and Kristoffer Lassen . 2019. “A Nationwide Cohort Study of Resection Rates and Short‐Term Outcomes in Open and Laparoscopic Distal Pancreatectomy.” International Hepato‐Pancreato‐Biliary Association 21(6): 669–678. 10.1016/j.hpb.2018.10.006. 30391219

[6] Seldomridge, Ashlee N. , Gordana Rasic , Marianna V. Papageorge , Sing Chau Ng , Susanna W. L. de Geus , Alison P. Woods , David McAneny , Jennifer F. Tseng , and Teviah E. Sachs . 2024. “Trends in Access to Minimally Invasive Pancreaticoduodenectomy for Pancreatic Cancers.” International Hepato‐Pancreato‐Biliary Association 26(3): 333–343. 10.1016/j.hpb.2023.11.012. 38087704

[7] van Hilst, Jony , Thijs de Rooij , Koop Bosscha , David J. Brinkman , Susan van Dieren , Marcel G. Dijkgraaf , Michael F. Gerhards , et al. 2019. “Laparoscopic versus Open Pancreatoduodenectomy for Pancreatic or Periampullary Tumours (LEOPARD‐2): a Multicentre, Patient‐Blinded, Randomised Controlled Phase 2/3 Trial.” Lancet Gastroenterol Hepatol 4(3): 199–207. 10.1016/S2468-1253(19)30004-4. 30685489

[8] van Hilst, Jony , Thijs de Rooij , Sjors Klompmaker , Majd Rawashdeh , Francesca Aleotti , Bilal Al‐Sarireh , Adnan Alseidi , et al. 2019. “Minimally Invasive versus Open Distal Pancreatectomy for Ductal Adenocarcinoma (DIPLOMA): A Pan‐European Propensity Score Matched Study.” Annals of Surgery 269(1): 10–17. 10.1097/SLA.0000000000002561. 29099399

[9] Girgis, Mark D. , Mazen S. Zenati , Jonathan C. King , Ahmad Hamad , Amer H. Zureikat , Herbert J. Zeh , and Melissa E. Hogg . 2021. “Oncologic Outcomes after Robotic Pancreatic Resections Are Not Inferior to Open Surgery.” Annals of Surgery 274(3): E262–E268. 10.1097/SLA.0000000000003615. 31663967

[10] Nassour, Ibrahim , Sam C. Wang , Alana Christie , Mathew M. Augustine , Matthew R. Porembka , Adam C. Yopp , Michael A. Choti , et al. 2018. “Minimally Invasive versus Open Pancreaticoduodenectomy.” Annals of Surgery 268(1): 151–157. 10.1097/SLA.0000000000002259. 28486387

[11] Wach, Michael M. , Ajay A. Myneni , Lorin Miller , Joseph Boccardo , Irada Ibrahim‐Zada , Steven S. Schwaitzberg , Katia Noyes , and Csaba Gajdos . 2022. “An Assessment of Perioperative Outcomes for Open, Laparoscopic, and Robot‐Assisted Pancreaticoduodenectomy in New York State.” Journal of Surgical Oncology 126(8): 1434–1441: Published online. 10.1002/jso.27075. 35986891

[12] Watson, Michael D. , Maria R. Baimas‐George , Kyle J. Thompson , David A. Iannitti , Lee M. Ocuin , Erin H. Baker , John B. Martinie , and Dionisios Vrochides . 2020. “Improved Oncologic Outcomes for Minimally Invasive Left Pancreatectomy: Propensity‐Score Matched Analysis of the National Cancer Database.” Journal of Surgical Oncology 122(7): 1383–1392. 10.1002/jso.26147. 32772366

[13] Baimas‐George, Maria , Michael Watson , Keith J. Murphy , David Iannitti , Erin Baker , Lee Ocuin , Dionisios Vrochides , and John B. Martinie . 2020. “Robotic Pancreaticoduodenectomy May Offer Improved Oncologic Outcomes over Open Surgery: a Propensity‐Matched Single‐Institution Study.” Surgical Endoscopy 34(8): 3644–3649. 10.1007/s00464-020-07564-x. 32328825

[14] Fisher, Alexander V. , Sara Fernandes‐Taylor , Jessica R. Schumacher , Jeffrey A. Havlena , Xing Wang , Elise H. Lawson , Sean M. Ronnekleiv‐Kelly , Emily R. Winslow , Sharon M. Weber , and Daniel E. Abbott . 2019. “Analysis of 90‐day Cost for Open versus Minimally Invasive Distal Pancreatectomy.” International Hepato‐Pancreato‐Biliary Association 21(1): 60–66. 10.1016/j.hpb.2018.07.003. 30076011

[15] Benzing, Christian , Lea Timmermann , Thomas Winklmann , Lena Marie Haiden , Karl Herbert Hillebrandt , Axel Winter , Max Magnus Maurer , et al. 2022. “Robotic versus Open Pancreatic Surgery: a Propensity Score‐Matched Cost‐Effectiveness Analysis.” Langenbeck's Archives of Surgery 407(5): 1923–1933. 10.1007/s00423-022-02471-2. PMC9399018 35312854

[16] Premier Healthcare Database . March 2, 2020. “White Paper: Data that Informs and Performs.”. https://learn.premierinc.com/white‐papers/premier‐healthcare‐database‐whitepaper. Accessed, May 17, 2023.

[17] v o n Elm, Erik , Douglas G. Altman , Matthias Egger , Stuart J. Pocock , Peter C. Gøtzsche , and Jan P. Vandenbroucke . 2007. “The Strengthening the Reporting of Observational Studies in Epidemiology (STROBE) Statement: Guidelines for Reporting Observational Studies.” Lancet 370(9596): 1453–1457. 10.1016/S0140-6736(07)61602-X. 18064739

[18] Jiang, Jerry , Alex Upfill‐Brown , Amanda M. Dann , Stephanie S. Kim , Mark D. Girgis , Jonathan C. King , and Timothy R. Donahue . 2019. “Association of Hospital Length of Stay and Complications with Readmission after Open Pancreaticoduodenectomy.” JAMA Surg 154(1): 88. 10.1001/jamasurg.2018.3213. 30325979 PMC6439846

[19] Björnsson, B. , A. Lindhoff Larsson , C. Hjalmarsson , T. Gasslander , and P. Sandström . 2020. “Comparison of the Duration of Hospital Stay after Laparoscopic or Open Distal Pancreatectomy: Randomized Controlled Trial.” British Journal of Surgery 107(10): 1281–1288. 10.1002/bjs.11554. 32259297

[20] Timmerhuis, Hester C. , Rejoice F. Ngongoni , Christopher Jensen , Michael Baiocchi , Jonathan C. DeLong , Monica M. Dua , Jeffrey A. Norton , George A. Poultsides , Patrick J. Worth , and Brendan C. Visser . 2022. “Comparison of Spleen‐Preservation vs Splenectomy in Minimally Invasive Distal Pancreatectomy: A Propensity‐Matched Analysis.” Journal of the American College of Surgeons 235(5): S52. 10.1097/01.XCS.0000896108.98531.49. 37653153

[21] Lof, S. , N. van der Heijde , M. Abuawwad , B. Al‐Sarireh , U. Boggi , G. Butturini , G. Capretti , et al. 2021. “Robotic versus Laparoscopic Distal Pancreatectomy: Multicentre Analysis.” British Journal of Surgery 108(2): 188–195. 10.1093/bjs/znaa039. 33711145

[22] Panni, Roheena Z. , Usman Y. Panni , Jingxia Liu , Gregory A. Williams , Ryan C. Fields , Dominic E. Sanford , William G. Hawkins , and Chet W. Hammill . 2021. “Re‐defining a High Volume Center for Pancreaticoduodenectomy.” International Hepato‐Pancreato‐Biliary Association 23(5): 733–738. 10.1016/j.hpb.2020.09.009. 32994102

[23] Sharpe, Susan M. , Mark S. Talamonti , Chihsiung E. Wang , Richard A. Prinz , Kevin K. Roggin , David J. Bentrem , David J. Winchester , Robert D. W. Marsh , Susan J. Stocker , and Marshall S. Baker . 2015. “Early National Experience with Laparoscopic Pancreaticoduodenectomy for Ductal Adenocarcinoma: A Comparison of Laparoscopic Pancreaticoduodenectomy and Open Pancreaticoduodenectomy from the National Cancer Data Base.” Journal of the American College of Surgeons 221(1): 175–184. 10.1016/j.jamcollsurg.2015.04.021. 26095569

[24] Kutlu, Onur C. , Eduardo A. Vega , Omid Salehi , Christopher Lathan , Sunhee Kim , Sandeep Krishnan , Christopher Stallwood , Olga Kozyreva , and Claudius Conrad . 2021. “Laparoscopic Pancreatectomy for Cancer in High Volume Centers Is Associated with an Increased Use and Fewer Delays of Adjuvant Chemotherapy.” International Hepato‐Pancreato‐Biliary Association 23(4): 625–632. 10.1016/j.hpb.2020.09.003. 32988752

[25] Panni, R. Z. , X. Lu , C. Hammill , R. C. Fields , W. G. Hawkins , and D. E. Sanford . 2020. “Defining High Volume Center for Distal Pancreatectomy.” International Hepato‐Pancreato‐Biliary Association 22: S100–S101. 10.1016/j.hpb.2020.04.591. 32994102

[26] Müller, P. C. , C. Kuemmerli , A. Cizmic , S. Sinz , P. Probst , M. de Santibanes , S. V. Shrikhande , et al. 2022. “Learning Curves in Open, Laparoscopic, and Robotic Pancreatic Surgery.” Annals of Surgery Open 3(1): e111. 10.1097/as9.0000000000000111. 37600094 PMC10431463

[27] Kim, Song C. , Ki B. Song , Yong S. Jung , Young H. Kim , Do H. Park , Sang S. Lee , Dong W. Seo , et al. 2013. “Short‐term Clinical Outcomes for 100 Consecutive Cases of Laparoscopic Pylorus‐Preserving Pancreatoduodenectomy: Improvement with Surgical Experience.” Surgical Endoscopy 27(1): 95–103. 10.1007/s00464-012-2427-9. 22752284

[28] Fung, Gayle , Menazir Sha , Basir Kunduzi , Farid Froghi , Saad Rehman , and Saied Froghi . 2022. “Learning Curves in Minimally Invasive Pancreatic Surgery: a Systematic Review.” Langenbeck's Archives of Surgery 407(6): 2217–2232. 10.1007/s00423-022-02470-3. PMC9467952 35278112

[29] Khachfe, Hussein H. , Ibrahim Nassour , Abdulrahman Y. Hammad , Jacob C. Hodges , Samer AlMasri , Hao Liu , Anissa deSilva , et al. 2023. “Robotic Pancreaticoduodenectomy: Increased Adoption and Improved Outcomes ‐ Is Laparoscopy Still Justified?”. Annals of Surgery 278(3): e563–e569: Published online. 10.1097/SLA.0000000000005687, August 24, 2022. 36000753 PMC11186698

[30] Wach, Michael M. , Ajay A. Myneni , Lorin Miller , Joseph Boccardo , Irada Ibrahim‐Zada , Steven S. Schwaitzberg , Katia Noyes , and Csaba Gajdos . 2022. “An Assessment of Perioperative Outcomes for Open, Laparoscopic, and Robot‐Assisted Pancreaticoduodenectomy in New York State.” Journal of Surgical Oncology 126(8): 1434–1441. 10.1002/jso.27075. 35986891

[31] Zureikat, Amer H. , Lauren M. Postlewait , Yuan Liu , Theresa W. Gillespie , Sharon M. Weber , Daniel E. Abbott , Syed A. Ahmad , et al. 2016. “A Multi‐Institutional Comparison of Perioperative Outcomes of Robotic and Open Pancreaticoduodenectomy.” Annals of Surgery 264(4): 640–649: Lippincott Williams and Wilkins. 10.1097/SLA.0000000000001869. 27433907

[32] Nassour, Ibrahim , Sam C. Wang , Alana Christie , Mathew M. Augustine , Matthew R. Porembka , Adam C. Yopp , Michael A. Choti , et al. 2018. “Minimally Invasive versus Open Pancreaticoduodenectomy.” Annals of Surgery 268(1): 151–157. 10.1097/SLA.0000000000002259. 28486387

[33] Poves, Ignasi , Fernando Burdío , Olga Morató , Mar Iglesias , Aleksander Radosevic , Lucas Ilzarbe , Laura Visa , and Luís Grande . 2018. “Comparison of Perioperative Outcomes between Laparoscopic and Open Approach for Pancreatoduodenectomy: The Padulap Randomized Controlled Trial.” Annals of Surgery 268(5): 731–739: Lippincott Williams and Wilkins. 10.1097/SLA.0000000000002893. 30138162

[34] Sharpe, Susan M. , Mark S. Talamonti , Chihsiung E. Wang , Richard A. Prinz , Kevin K. Roggin , David J. Bentrem , David J. Winchester , Robert D. W. Marsh , Susan J. Stocker , and Marshall S. Baker . 2015. “Early National Experience with Laparoscopic Pancreaticoduodenectomy for Ductal Adenocarcinoma: A Comparison of Laparoscopic Pancreaticoduodenectomy and Open Pancreaticoduodenectomy from the National Cancer Data Base.” Journal of the American College of Surgeons 221(1): 175–184. 10.1016/j.jamcollsurg.2015.04.021. 26095569

[35] Nassour, Ibrahim , Sam C. Wang , Matthew R. Porembka , Adam C. Yopp , Michael A. Choti , Mathew M. Augustine , Patricio M. Polanco , John C. Mansour , and Rebecca M. Minter . 2017. “Robotic versus Laparoscopic Pancreaticoduodenectomy: a NSQIP Analysis.” Journal of Gastrointestinal Surgery 21(11): 1784–1792. 10.1007/s11605-017-3543-6. 28819886 PMC5789456

[36] Torphy, Robert J. , Chloe Friedman , Alison Halpern , Brandon C. Chapman , Steven S. Ahrendt , Martin M. McCarter , Barish H. Edil , Richard D. Schulick , and Ana Gleisner . 2019. “Comparing Short‐Term and Oncologic Outcomes of Minimally Invasive versus Open Pancreaticoduodenectomy across Low and High Volume Centers.” Annals of Surgery 270(6): 1147–1155. 10.1097/SLA.0000000000002810. 29771723

[37] Kim, Hyeyeon , Sung Hoon Choi , Jae Young Jang , Munseok Choi , Jae Hoon Lee , and Chang Moo Kang . 2022. “Multicenter Comparison of Totally Laparoscopic and Totally Robotic Pancreaticoduodenectomy: Propensity Score and Learning Curve‐matching Analyses.” Journal of Hepatobiliary Pancreat Science 29(3): 311–321. 10.1002/jhbp.1078. 34773395

[38] Zhou, Jiangjiao , Li Xiong , Xiongying Miao , Juan Liu , Heng Zou , and Yu Wen . 2020. “Outcome of Robot‐Assisted Pancreaticoduodenectomy during Initial Learning Curve versus Laparotomy.” Scientific Reports 10(1): 9621. 10.1038/s41598-020-66722-2. 32541683 PMC7295787

[39] Tran Cao, Hop S. , Nicole Lopez , David C. Chang , Andrew M. Lowy , Michael Bouvet , Joel M. Baumgartner , Mark A. Talamini , and Jason K. Sicklick . 2014. “Improved Perioperative Outcomes with Minimally Invasive Distal Pancreatectomy: Results from a Population‐Based Analysis.” JAMA Surg 149(3): 237–243. 10.1001/jamasurg.2013.3202. 24402232 PMC4383084

[40] de Rooij, Thijs , Jony van Hilst , Hjalmar van Santvoort , Djamila Boerma , Peter van den Boezem , Freek Daams , Ronald van Dam , et al. 2019. “Minimally Invasive versus Open Distal Pancreatectomy (LEOPARD): A Multicenter Patient‐Blinded Randomized Controlled Trial.” Annals of Surgery 269(1): 2–9. 10.1097/SLA.0000000000002979. 30080726

[41] Kwon, Jaewoo , Jae Hoon Lee , Seo Young Park , Yejong Park , Woohyung Lee , Ki Byung Song , Dae Wook Hwang , and Song Cheol Kim . 2022. “A Comparison of Robotic versus Laparoscopic Distal Pancreatectomy: Propensity Score Matching Analysis.” International Journal of Medical Robotics and Computer Assisted Surgery 18(2). 10.1002/rcs.2347. 34726827

[42] Kwon, Jaewoo , Seo Young Park , Yejong Park , Eunsung Jun , Woohyung Lee , Ki Byung Song , Jae Hoon Lee , Dae Wook Hwang , and Song Cheol Kim . 2021. “A Comparison of Minimally Invasive vs Open Distal Pancreatectomy for Resectable Pancreatic Ductal Adenocarcinoma: Propensity Score Matching Analysis.” Journal of Hepatobiliary Pancreat Science 28(11): 967–982. 10.1002/jhbp.853. 33091208

[43] De Pastena, Matteo , Alessandro Esposito , Salvatore Paiella , Niccolò Surci , Greta Montagnini , Giovanni Marchegiani , Giuseppe Malleo , et al. 2021. “Cost‐effectiveness and Quality of Life Analysis of Laparoscopic and Robotic Distal Pancreatectomy: a Propensity Score‐Matched Study.” Surgical Endoscopy 35(3): 1420–1428. 10.1007/s00464-020-07528-1. 32240383

[44] Statista . 2023. “Distribution of U.S, Healthcare from 2015‐2023, by Payer.”. https://www.statista.com/statistics/237043/us‐health‐care‐spending‐distribution/. Accessed, September 11, 2024.

[45] Premier Inc Applied Sciences . 2022. “PINC AI Healthcare Database: Data that Informs and Performs.”. https://offers.premierinc.com/rs/381‐NBB‐525/images/Premier‐Healthcare‐Database‐.

