# Supplementary material for: Current landscape of minimally invasive pancreatectomy for neoplasms: A retrospective cohort study
Source: World J Surg. 2024 Nov 22;49(1):241–52. doi: 10.1002/wjs.12408 (PMC11711114; doi:10.1002/wjs.12408)
Supplement: Supplementary file 1 — Supporting Information 1 [file WJS-49-241-s001.docx]

Supplementary Table 1. Procedure and Diagnosis Codes

| **Pancreaticoduodenectomy (PD)** | | |
| --- | --- | --- |
| ICD 9 | 52.7 | Radical pancreaticoduodenectomy |
| CPT | 48150 | Pancreatectomy, proximal subtotal with total duodenectomy, partial gastrectomy, choledochoenterostomy and gastrojejunostomy (Whipple-type procedure); with pancreatojejunostomy |
| CPT | 48152 | Pancreatectomy, proximal subtotal with total duodenectomy, partial gastrectomy, choledochoenterostomy and gastrojejunostomy (Whipple-type procedure); without pancreatojejunostomy |
| CPT | 48153 | Pancreatectomy, proximal subtotal with near-total duodenectomy, choledochoenterostomy and duodenojejunostomy (pylorus-sparing, Whipple- type procedure); with pancreatojejunostomy |
| CPT | 48154 | Pancreatectomy, proximal subtotal with near-total duodenectomy, choledochoenterostomy and duodenojejunostomy (pylorus-sparing, Whipple- type procedure); without pancreatojejunostomy |
| **ICD 10 Excision of pancreas + ANY (Duodenal surgery, Stomach surgery)** | | |
| Excision of pancreas | | |
| ICD 10 | 0FBG0ZZ | Excision of pancreas, open approach |
| ICD 10 | 0FBG4ZZ | Excision of pancreas, percutaneous endoscopic approach |
| Duodenal surgery or Stomach surgery | | |
| ICD 10 | 0DB90ZZ | Excision of Duodenum, Open Approach |
| ICD 10 | 0DB93ZZ | Excision of Duodenum, Percutaneous Approach |
| ICD 10 | 0DT90ZZ | Resection of Duodenum, Open Approach |
| ICD 10 | 0DT94ZZ | Resection of Duodenum, Percutaneous Endoscopic Approach |
| ICD 10 | 0DB60ZZ | Excision of Stomach, Open Approach |
| ICD 10 | 0DB64ZZ | Excision of Stomach, Percutaneous Endoscopic Approach |
| ICD 10 | 0DT70ZZ | Resection of Stomach, Pylorus, Open Approach |
| ICD 10 | 0DT74ZZ | Resection of Stomach, Pylorus, Percutaneous Endoscopic Approach |
| **Non-Pancreaticoduodenectomy Resections (non-PD)** | | |
| ICD 9 | 52.51 | Proximal pancreatectomy |
| ICD 9 | 52.52 | Distal pancreatectomy |
| ICD 9 | 52.53 | Radical subtotal pancreatectomy |
| ICD 9 | 52.59 | Other partial pancreatectomy |
| CPT | 48140 | Pancreatectomy, distal subtotal, with or without splenectomy; without pancreatojejunostomy |
| CPT | 48145 | Pancreatectomy, distal subtotal, with or without splenectomy; with pancreatojejunostomy |
| CPT | 48146 | Pancreatectomy, distal, near-total with preservation of duodenum (Child-type procedure) |
| **ICD 10 Excision of pancreas WITHOUT (Duodenal surgery, Stomach surgery)** | | |
| **Malignant neoplasm** | | |
| ICD 9 | 157.0 | Malignant neoplasm of head of pancreas |
| ICD 9 | 157.1 | Malignant neoplasm of body of pancreas |
| ICD 9 | 157.2 | Malignant neoplasm of tail of pancreas |
| ICD 9 | 157.3 | Malignant neoplasm of pancreatic duct |
| ICD 9 | 157.4 | Malignant neoplasm of islets of langerhans |
| ICD 9 | 157.8 | Malignant neoplasm of other specified sites of pancreas |
| ICD 9 | 157.9 | Malignant neoplasm of pancreas, part unspecified |
| ICD 10 | C25.0 | Malignant neoplasm of head of pancreas |
| ICD 10 | C25.1 | Malignant neoplasm of body of pancreas |
| ICD 10 | C25.2 | Malignant neoplasm of tail of pancreas |
| ICD 10 | C25.3 | Malignant neoplasm of pancreatic duct |
| ICD 10 | C25.4 | Malignant neoplasm of endocrine pancreas |
| ICD 10 | C25.7 | Malignant neoplasm of other parts of pancreas |
| ICD 10 | C25.8 | Malignant neoplasm of overlapping sites of pancreas |
| ICD 10 | C25.9 | Malignant neoplasm of pancreas, unspecified |
| ICD 9 | 152.0 | Malignant neoplasm of duodenum |
| ICD 9 | 156.1 | Malignant neoplasm of extrahepatic bile ducts |
| ICD 9 | 156.2 | Malignant neoplasm of ampulla of Vater |
| ICD 9 | 155.1 | Malignant neoplasm of intrahepatic bile ducts |
| ICD 9 | 158.0 | Malignant neoplasm of retroperitoneum |
| ICD 9 | 151.9 | Malignant neoplasm of stomach, unspecified site |
| ICD 9 | 171.5 | Malignant neoplasm of connective and other soft tissue of abdomen |
| ICD 9 | 209.29 | Malignant carcinoid tumor of other sites |
| ICD 9 | 209.30 | Malignant poorly differentiated neuroendocrine carcinoma, any site |
| ICD 9 | 209.30 | Malignant poorly differentiated neuroendocrine carcinoma, any site |
| ICD 9 | 209.01 | Malignant carcinoid tumor of the duodenum |
| ICD 9 | 230.9 | Carcinoma in situ of other and unspecified digestive organs |
| ICD 9 | 230.8 | Carcinoma in situ of liver and biliary system |
| ICD 10 | C17.0 | Malignant neoplasm of duodenum |
| ICD 10 | C24.0 | Malignant neoplasm of extrahepatic bile duct |
| ICD 10 | C24.1 | Malignant neoplasm of ampulla of Vater |
| ICD 10 | C22.1 | Intrahepatic bile duct carcinoma |
| ICD 10 | C48.0 | Malignant neoplasm of retroperitoneum |
| ICD 10 | C16.9 | Malignant neoplasm of stomach, unspecified |
| ICD 10 | C49.A2 | Gastrointestinal stromal tumor of stomach |
| ICD 10 | C7A.098 | Malignant carcinoid tumors of other sites |
| ICD 10 | C7A.1 | Malignant poorly differentiated neuroendocrine tumors |
| ICD 10 | C7A.8 | Other malignant neuroendocrine tumors |
| ICD 10 | C7A.010 | Malignant carcinoid tumor of the duodenum |
| ICD 10 | D01.7 | Carcinoma in situ of other specified digestive organs |
| ICD 10 | D01.5 | Carcinoma in situ of liver, gallbladder and bile ducts |
| ICD 9 | 197.8 | Secondary malignant neoplasm of other digestive organs and spleen |
| ICD 9 | 197.6 | Secondary malignant neoplasm of retroperitoneum and peritoneum |
| ICD 9 | 197.7 | Malignant neoplasm of liver, secondary |
| ICD 10 | C78.89 | Secondary malignant neoplasm of other digestive organs |
| ICD 10 | C78.6 | Secondary malignant neoplasm of retroperitoneum and peritoneum |
| ICD 10 | C78.7 | Secondary malignant neoplasm of liver and intrahepatic bile duct |
| **Benign neoplasm** | | |
| ICD 9 | 211.6 | Benign neoplasm of pancreas, except islets of Langerhans |
| ICD 9 | 211.7 | Benign neoplasm of islets of Langerhans |
| ICD 10 | D13.6 | Benign neoplasm of pancreas |
| ICD 10 | D13.7 | Benign neoplasm of endocrine pancreas |
| ICD 9 | 211.2 | Benign neoplasm of duodenum, jejunum, and ileum |
| ICD 9 | 211.5 | Benign neoplasm of liver and biliary passages |
| ICD 9 | 209.69 | Benign carcinoid tumor of other sites |
| ICD 9 | 235.3 | Neoplasm of uncertain behavior of liver and biliary passages |
| ICD 9 | 239.0 | Neoplasm of unspecified nature of digestive system |
| ICD 9 | 235.5 | Neoplasm of uncertain behavior of other and unspecified digestive organs |
| ICD 10 | D13.2 | Benign neoplasm of duodenum |
| ICD 10 | D13.5 | Benign neoplasm of extrahepatic bile ducts |
| ICD 10 | D3A.098 | Benign carcinoid tumors of other sites |
| ICD 10 | D37.6 | Neoplasm of uncertain behavior of liver, gallbladder and bile ducts |
| ICD 10 | D49.0 | Neoplasm of unspecified behavior of digestive system |
| ICD 10 | D37.8 | Neoplasm of uncertain behavior of other specified digestive organs |

ICD, International Classification of Disease; CPT, Current Procedural Terminology

Supplementary Table 2: Trend Utilization of minimally invasive pancreatectomy among Hospitals- stratified by hospital volume

|  | High volume hospital | | |  | Low volume hospital | | |
| --- | --- | --- | --- | --- | --- | --- | --- |
| Year | Total N | MIS, N | MIS, % |  | Total N | MIS, N | MIS, % |
| PD (Cutoff: 9 cases/year) | | |  |  |  |  |  |
| 2013 | 28 | 12 | 42.9% |  | 143 | 32 | 22.4% |
| 2014 | 41 | 22 | 53.7% |  | 129 | 32 | 24.8% |
| 2015 | 37 | 24 | 64.9% |  | 133 | 36 | 27.1% |
| 2016 | 47 | 18 | 38.3% |  | 126 | 21 | 16.7% |
| 2017 | 50 | 27 | 54.0% |  | 132 | 25 | 18.9% |
| 2018 | 61 | 35 | 57.4% |  | 113 | 28 | 24.8% |
| 2019 | 56 | 37 | 66.1% |  | 111 | 21 | 18.9% |
| 2020 | 47 | 31 | 66.0% |  | 99 | 25 | 25.3% |
| Non-PD (Cutoff: 5 cases/year) | | |  |  |  |  |  |
| 2013 | 26 | 21 | 80.8% |  | 132 | 45 | 34.1% |
| 2014 | 32 | 24 | 75.0% |  | 123 | 38 | 30.9% |
| 2015 | 35 | 31 | 88.6% |  | 126 | 63 | 50.0% |
| 2016 | 45 | 39 | 86.7% |  | 105 | 71 | 67.6% |
| 2017 | 54 | 48 | 88.9% |  | 119 | 79 | 66.4% |
| 2018 | 51 | 48 | 94.1% |  | 110 | 73 | 66.4% |
| 2019 | 59 | 54 | 91.5% |  | 105 | 62 | 59.0% |
| 2020 | 60 | 57 | 95.0% |  | 80 | 52 | 65.0% |

PD, pancreaticoduodenectomy; Non-PD, non-pancreaticoduodenectomy resections; MIS, minimally invasive surgery

Supplementary Table 3. Association of Surgical Approach and Postoperative Outcomes for Pancreaticoduodenectomy (PD)

| **Variable** | **Type of Procedure** | | |  | **RS vs. LS** | |  |
| --- | --- | --- | --- | --- | --- | --- | --- |
|  | **Open**, N = 8,663 | **LS**, N = 593 | **RS**, N = 607 |  | **Diff/OR (95%CI)** | **P value** | |
| **LOS** |  |  |  |  |  |  | |
| Mean (SD) | 11.3 (9.5) | 11.2 (9.2) | 10.2 (10.9) |  | -1.0 (-1.9 to 0.02) | 0.056 | |
| Median (Q1, Q3) | 8 (6, 13) | 8 (6, 13) | 7 (5, 11) |  |  |  | |
| **LOS ≥14 days** | 2,032 (23.5%) | 141 (23.8%) | 100 (16.5%) |  | 0.66 (0.49 to 0.88) | 0.005 | |
| **OR Time** |  |  |  |  |  |  | |
| Mean (SD) | 440.0 (350.0) | 479.6 (170.2) | 530.4 (199.9) |  | 65 (31 to 100) | <0.001 | |
| Median (Q1, Q3) | 407 (330, 510) | 465 (360, 555) | 480 (405, 594) |  |  |  | |
| **ICU admission** | 5,499 (63.5%) | 357 (60.2%) | 371 (61.1%) |  | 0.30 (0.23 to 0.40) | <0.001 | |
| **In-hospital mortality** | 248 (2.9%) | 21 (3.5%) | 18 (3.0%) |  | 1.00 (0.79 to 1.27) | 0.981 | |
| **Discharge home** | 6,994 (80.7%) | 470 (79.3%) | 482 (79.4%) |  | 0.86 (0.44 to 1.65) | 0.646 | |
| **In-hospital reoperation** | 457 (5.3%) | 39 (6.6%) | 40 (6.6%) |  | 1.01 (0.75 to 1.36) | 0.930 | |
| **In hospital Complication** | 3,999 (46.2%) | 296 (49.9%) | 285 (47.0%) |  | 1.09 (0.68 to 1.74) | 0.723 | |
| Intraoperative | 182 (2.1%) | 20 (3.4%) | 15 (2.5%) |  | 0.87 (0.69 to 1.10) | 0.232 | |
| Pulmonary | 1,123 (13.0%) | 83 (14.0%) | 99 (16.3%) |  | 0.82 (0.40 to 1.62) | 0.562 | |
| Mechanical ventilation | 638 (7.4%) | 57 (9.6%) | 50 (8.2%) |  | 1.17 (0.85 to 1.63) | 0.334 | |
| Transfusion | 1,674 (19.3%) | 149 (25.1%) | 136 (22.4%) |  | 0.88 (0.58 to 1.33) | 0.547 | |
| Bleeding | 2,284 (26.4%) | 172 (29.0%) | 174 (28.7%) |  | 0.93 (0.71 to 1.22) | 0.585 | |
| Infections | 1,243 (14.3%) | 90 (15.2%) | 86 (14.2%) |  | 0.90 (0.70 to 1.17) | 0.432 | |
| Venous thromboembolism | 180 (2.1%) | 13 (2.2%) | 14 (2.3%) |  | 0.94 (0.68 to 1.31) | 0.724 | |
| **30-day Readmission** | 1,592 (18.4%) | 103 (17.4%) | 123 (20.3%) |  | 1.11 (0.51 to 2.44) | 0.791 | |
| **30-day Perioperative cost** |  |  |  |  | 1.14 (0.85 to 1.53) | 0.392 | |
| Mean (SD) | 48,039 (40,936) | 51,063 (47,613) | 54,381 (44,067) |  | 4,691 (-8.7 to 9,413) | 0.052 | |
| Median (Q1, Q3) | 37,330 (27,847, 53,371) | 38,061 (29,192, 55,651) | 43,083 (32,760, 58,189) |  |  |  | |

All measures of association presented were adjusted for patient and hospital characteristics, as described in the methods section.

LS, laparoscopic surgery; RS, robotic-assisted surgery; Diff, mean difference; OR, odds ratio; CI, confidence interval; LOS, length of stay; OR time, operative room time; ICU, intensive care unit.

Supplementary Table 4. Association of Surgical Approach and Postoperative Outcomes for Non-Pancreaticoduodenectomy Resections (Non-PD)

| **Variable** | **Type of Procedure** | | |  | **RS vs. LS** | |
| --- | --- | --- | --- | --- | --- | --- |
|  | **Open**, N = 2,678 | **LS**, N = 1,347 | **RS**, N = 1,170 |  | **OR/Diff (95%CI)** | **P value** |
| **LOS** |  |  |  |  |  |  |
| Mean (SD) | 7.4 (5.7) | 6.2 (5.1) | 6.1 (5.2) |  | -0.08 (-0.43 to 0.27) | 0.647 |
| Median (Q1, Q3) | 6 (5, 8) | 5 (4, 7) | 5 (4, 7) |  |  |  |
| **LOS ≥10 days** | 435 (16.2%) | 162 (12.0%) | 111 (9.5%) |  | 0.78 (0.60 to 1.02) | 0.071 |
| **OR Time** |  |  |  |  |  |  |
| Mean (SD) | 294.8 (195.1) | 298.4 (155.2) | 384.2 (188.9) |  | 82 (66 to 97) | <0.001 |
| Median (Q1, Q3) | 267 (210, 345) | 270 (210, 360) | 339 (261, 450) |  |  |  |
| **Splenectomy** | 1,816 (67.8%) | 907 (67.3%) | 691 (59.1%) |  | 0.42 (0.33 to 0.54) | <0.001 |
| **ICU admission** | 1,089 (40.7%) | 353 (26.2%) | 280 (23.9%) |  | 0.67 (0.57 to 0.79) | <0.001 |
| **Index mortality** | 34 (1.3%) | 10 (0.7%) | 12 (1.0%) |  | 0.87 (0.72 to 1.05) | 0.140 |
| **Discharge home** | 2,397 (89.5%) | 1,230 (91.3%) | 1,062 (90.8%) |  | 1.35 (0.57 to 3.28) | 0.490 |
| **Index reoperation** | 79 (2.9%) | 30 (2.2%) | 31 (2.6%) |  | 0.89 (0.67 to 1.20) | 0.451 |
| **In-hospital Complication** | 855 (31.9%) | 320 (23.8%) | 294 (25.1%) |  | 1.29 (0.77 to 2.18) | 0.331 |
| Intraoperative | 48 (1.8%) | 22 (1.6%) | 16 (1.4%) |  | 1.07 (0.88 to 1.29) | 0.493 |
| Pulmonary | 234 (8.7%) | 87 (6.5%) | 85 (7.3%) |  | 0.91 (0.46 to 1.75) | 0.779 |
| Mechanical ventilation | 113 (4.2%) | 44 (3.3%) | 46 (3.9%) |  | 1.15 (0.84 to 1.58) | 0.387 |
| Transfusion | 333 (12.4%) | 112 (8.3%) | 92 (7.9%) |  | 1.23 (0.80 to 1.90) | 0.353 |
| Bleeding | 473 (17.7%) | 165 (12.2%) | 173 (14.8%) |  | 0.99 (0.73 to 1.32) | 0.921 |
| Infections | 185 (6.9%) | 72 (5.3%) | 61 (5.2%) |  | 1.21 (0.96 to 1.54) | 0.112 |
| Venous thromboembolism | 32 (1.2%) | 6 (0.4%) | 11 (0.9%) |  | 0.94 (0.65 to 1.34) | 0.728 |
| **30-day Readmission** | 455 (17.0%) | 216 (16.0%) | 206 (17.6%) |  | 2.32 (0.87 to 6.82) | 0.102 |
| **30-day Perioperative cost** |  |  |  |  | 1.12 (0.91 to 1.39) | 0.287 |
| Mean (SD) | 32,291 (26,240) | 30,078 (23,585) | 36,519 (33,743) |  | 7,351 (5,444 to 9,291) | <0.001 |
| Median (Q1, Q3) | 25,481 (18,793, 36,360) | 23,819 (17,214, 33,571) | 28,940 (21,633, 40,200) |  |  |  |

All measures of association presented were adjusted for patient and hospital characteristics, as described in the methods section.

LS, laparoscopic surgery; RS, robotic-assisted surgery; OR, odds ratio; Diff, mean difference; CI, confidence interval; LOS, length of stay; OR time, operative room time; ICU, intensive care unit.

Supplementary Table 5. Impact of Conversion on Outcomes for Minimally invasive Pancreaticoduodenectomy (PD)

| **Variable** | **MIS** | **MIS-Converted** |  | **MIS-Converted vs MIS** | |
| --- | --- | --- | --- | --- | --- |
|  | N=796 | N = 404 |  | **Diff/OR (95%CI)** | **P value** |
| **LOS** |  |  |  |  |  |
| Mean (SD) | 10.6 (10.6) | 10.9 (9.0) |  | -0.10 (-1.2 to 1.1) | 0.864 |
| Median (Q1, Q3) | 7 (5, 11) | 8 (6, 13) |  |  |  |
| **Prolonged LOS (≥14 days)** | 153 (19.2%) | 88 (21.8%) |  | 0.95 (0.69 to 1.31) | 0.774 |
| **OR Time** |  |  |  |  |  |
| Mean (SD) | 507.4 (197.2) | 501.2 (166.7) |  | -2.5 (-24 to 19) | 0.819 |
| Median (Q1, Q3) | 480 (390, 570) | 480 (390, 570) |  |  |  |
| **ICU admission** | 494 (62.1%) | 234 (57.9%) |  | 0.84 (0.65 to 1.10) | 0.205 |
| **In-hospital mortality** | 25 (3.1%) | 14 (3.5%) |  | 0.99 (0.48 to 1.98) | 0.978 |
| **Discharge home** | 633 (79.5%) | 319 (79.0%) |  | 1.08 (0.79 to 1.50) | 0.630 |
| **In-hospital reoperation** | 53 (6.7%) | 26 (6.4%) |  | 0.83 (0.49 to 1.37) | 0.466 |
| **In-hospital Complication** | 371 (46.6%) | 210 (52.0%) |  | 1.17 (0.90 to 1.51) | 0.233 |
| Intraoperative | 18 (2.3%) | 17 (4.2%) |  | 2.11 (1.03 to 4.29) | 0.039 |
| Pulmonary, n (%) | 118 (14.8%) | 64 (15.8%) |  | 0.95 (0.67 to 1.35) | 0.791 |
| Mechanical ventilation | 63 (7.9%) | 44 (10.9%) |  | 1.17 (0.75 to 1.81) | 0.485 |
| Transfusion | 170 (21.4%) | 115 (28.5%) |  | 1.43 (1.07 to 1.91) | 0.017 |
| Bleeding | 218 (27.4%) | 128 (31.7%) |  | 1.18 (0.90 to 1.56) | 0.236 |
| Infections | 117 (14.7%) | 59 (14.6%) |  | 0.82 (0.57 to 1.18) | 0.292 |
| Venous thromboembolism | 15 (1.9%) | 12 (3.0%) |  | 1.52 (0.66 to 3.44) | 0.316 |
| **30-day Readmission** | 150 (18.8%) | 76 (18.8%) |  | 1.00 (0.72 to 1.37) | 0.986 |
| **30-day Perioperative cost** |  |  |  |  |  |
| Mean (SD) | 52,539.0 (47,333.1) | 53,140.3 (42,879.0) |  | -1,875 (-6,776 to 3,243) | 0.460 |
| Median (Q1, Q3) | 40,361 (31,043, 55,510) | 41,829 (30,382, 59,513) |  |  |  |

All measures of association presented were adjusted for patient and hospital characteristics, as described in the methods section.

MIS. Minimally invasive surgery; Diff, mean difference; OR, odds ratio; CI, confidence interval; LOS, length of stay; OR time, operative room time; ICU, intensive care unit.

Supplementary Table 6. Impact of Conversion on Outcomes for Minimally invasive Non-Pancreaticoduodenectomy Resections (Non-PD)

| **Variable** | **MIS** | **MIS-Converted** |  | **MIS-Converted vs MIS** | |
| --- | --- | --- | --- | --- | --- |
|  | N=2130 | N = 387 |  | **Diff/OR (95%CI)** | **P value** |
| **LOS** |  |  |  |  |  |
| Mean (SD) | 6.0 (5.1) | 7.3 (5.3) |  | 1.3 (0.71 to 1.9) | <0.001 |
| Median (Q1, Q3) | 5 (4, 6) | 6 (5, 8) |  |  |  |
| **Prolonged LOS (≥10 days)** | 211 (9.9%) | 62 (16.0%) |  | 1.65 (1.19 to 2.26) | 0.002 |
| **OR Time** |  |  |  |  |  |
| Mean (SD) | 338.3 (183.6) | 338.5 (134.3) |  | 6.1 (-11 to 24) | 0.492 |
| Median (Q1, Q3) | 300 (225, 390) | 300 (240, 405) |  |  |  |
| **Splenectomy** | 1,278 (60.0%) | 320 (82.7%) |  | 3.24 (2.46 to 4.32) | <0.001 |
| **ICU admission** | 476 (22.3%) | 157 (40.6%) |  | 2.47 (1.94 to 3.14) | <0.001 |
| **In-hospital mortality** | 17 (0.8%) | 5 (1.3%) |  | 1.39 (0.44 to 3.70) | 0.542 |
| **Discharge home** | 1,958 (91.9%) | 334 (86.3%) |  | 0.58 (0.41 to 0.83) | 0.003 |
| **In-hospital reoperation** | 50 (2.3%) | 11 (2.8%) |  | 1.32 (0.64 to 2.52) | 0.424 |
| **In-hospital Complication** | 455 (21.4%) | 159 (41.1%) |  | 2.57 (2.03 to 3.26) | <0.001 |
| Intraoperative | 16 (0.8%) | 22 (5.7%) |  | 9.09 (4.60 to 18.4) | <0.001 |
| Pulmonary, n (%) | 130 (6.1%) | 42 (10.9%) |  | 1.79 (1.21 to 2.59) | 0.003 |
| Mechanical ventilation | 70 (3.3%) | 20 (5.2%) |  | 1.58 (0.91 to 2.63) | 0.087 |
| Transfusion | 138 (6.5%) | 66 (17.1%) |  | 2.94 (2.11 to 4.06) | <0.001 |
| Bleeding | 241 (11.3%) | 97 (25.1%) |  | 2.81 (2.12 to 3.71) | <0.001 |
| Infections | 104 (4.9%) | 29 (7.5%) |  | 1.52 (0.97 to 2.33) | 0.060 |
| Venous thromboembolism | 15 (0.7%) | 2 (0.5%) |  | 0.81 (0.13 to 2.97) | 0.780 |
| **30-day Readmission** | 345 (16.2%) | 77 (19.9%) |  | 1.28 (0.96 to 1.69) | 0.085 |
| **30-day Perioperative cost** |  |  |  |  |  |
| Mean (SD) | 32,194.7 (28,689.2) | 37,901.9 (29,798.3) |  | 4,946 (2,061 to 8,080) | 0.001 |
| Median (Q1, Q3) | 25,953 (18,615, 36,033) | 28,756 (22,155, 42,778) |  |  |  |

All measures of association presented were adjusted for patient and hospital characteristics, as described in the methods section.

MIS. Minimally invasive surgery; Diff, mean difference; OR, odds ratio; CI, confidence interval; LOS, length of stay; OR time, operative room time; ICU, intensive care unit
